# A descriptive study on demographic and behavioral characteristics of males and their responses to a male involvement intervention in Blantyre, Malawi

**DOI:** 10.11604/pamj.2016.25.229.10014

**Published:** 2016-12-07

**Authors:** Alinane Linda Nyondo-Mipando, Angela Faith Chimwaza, Adamson Sinjani Muula

**Affiliations:** 1 School of Public Health and Family Medicine, College of Medicine University of Malawi, Malawi; 2 Malawi Liverpool Wellcome Trust Clinical Research Programme, Blantyre, Malawi, Malawi; 3 Kamuzu College of Nursing, University of Malawi, Malawi

**Keywords:** Male Involvement, PMTCT, behaviour, characteristics

## Abstract

**Introduction:**

Male involvement (MI) remains a key factor in the enrollment and retention of pregnant women in the Prevention of Mother to child transmission (PMTCT) of Human Immunodeficiency Virus (HIV) services. The objective of this study was to describe the characteristics of men who accompanied their partners for PMTCT services and secondly, describe the reported reasons for the non-reporting by men for the services in Blantyre, Malawi.

**Methods:**

All men included in this analysis were partners of pregnant women enrolled in a MI in PMTCT randomized controlled trial (RCT), which took place in Blantyre, Malawi from 14 June 2013 to 24 February 2014. After randomization women were asked to invite their male partners for PMTCT services either through an invitation card or word of mouth invite. Descriptive statistics were tabulated using Stata.

**Results:**

Of the 462 women randomized, 109 (23.59%) women came back to the clinic with their male partner following the intervention. The majority, 307 (66.5%) women returned to the clinic without their partners. Although most men accepted the intervention, some failed to accompany their partners because of work obligations, a lack of interest in accompanying their partners for the service, and others promised to report at the next clinic visit.

**Conclusion:**

The characteristics of men that reported were similar in the two groups, suggesting that demographic characteristics may not greatly influence their decision to be involved in PMTCT services. There is need to develop more flexible strategies to include men in PMTCT programmes.

## Introduction

Male involvement (MI) remains a key factor in the enrollment and retention of pregnant women in the Prevention of Mother to child transmission (PMTCT) of Human Immunodeficiency Virus (HIV) services [1–3]. In Malawi, relevance of male partner participation is more highlighted with the implementation of Option B+ as a PMTCT strategy because MI in PMTCT is significant for the uptake of HIV testing and antiretrovirals (ARVs) [3, 4]. Option B+, which is the policy for PMTCT services in Malawi since 2011, entails offering of triple antiretrovirals to a woman irrespective of CD4 count or clinical staging. Antiretroviral therapy is continued for life and the infant receives nevirapine daily, from birth until 4-6 weeks irrespective of the choice on infant feeding method [5]. Retention in care under Option B+ has been challenging as evidenced by preliminary assessments in the country which have indicated that more women who started on ARVs based on PMTCT needs were not retained in care than those who started on ARVs because of their clinical condition [6]. The lack of MI inevitably leads to withdrawals from an effective program and substantial losses at every step of the PMTCT cascade [7–9]. Although several strategies for the inclusion of male partners have been suggested for Malawi [10, 11] they have not resulted into improved rates of MI because they have not been implemented to scale. A review by Ditekemena on factors associated with MI showed that men who were more involved in the maternal and child health services were those who were older in age, in monogamous relationships and cohabiting partnerships; involvement was also associated with education and type of occupation; those with higher education and those in more paying jobs were more likely to be involved than their counterparts [12]. As Malawi aims at optimizing the uptake and retention of women in PMTCT programmes, understanding the characteristics of the men who accompany their partners, their response to implemented strategies and the hindrances to their involvement in PMTCT services remain important. This information is fundamental in optimizing interventions for men currently not participating in PMTCT services as well as strengthening the interventions for the men that participate. Currently, the rates for MI in MTCT and the entire maternal and child health services in Malawi remains low with self-reported rates by women ranging from 3.2% to 23% [3, 13, 14]. The purpose of this study was to describe the characteristics of men who accompanied their partners for PMTCT services and secondly, describe the reported reasons for the lack of participation by men in PMTCT services in Blantyre, Malawi.

## Methods

### Study design

All men included in this analysis were partners of pregnant women enrolled in a MI in PMTCT randomized controlled trial (RCT) which took place in Blantyre, Malawi from 14 June 2013 to 24 February 2014. The study design, eligibility, interventions, procedures and primary findings have been reported in another article [15]. Briefly, the trial recruited pregnant women of less than or equal to 30 weeks gestation and determined the efficacy of an invitation card as a strategy for inviting male partners to antenatal care. In the RCT the study intervention was an invitation card to a pregnant woman’s partner while women in the standard of care group delivered a “word of mouth invite” as a strategy of inviting their male partners to accompany them for PMTCT at the next study scheduled visit. The women had two follow up visits, 2 and 6 weeks after recruitment [15]. In this article, we describe the baseline characteristics of men that reported for PMTCT with their partners following the intervention. We also describe the reasons for the lack of participation of male partners in PMTCT services following invitations, as reported by their female partners.

### Study setting and population and sample size

The study was conducted at South Lunzu and Mpemba Health Centres in Blantyre, Malawi. Both health centers offer PMTCT services within their antenatal care service and serve a semi urban population [15]. The study included all men whose partners participated in the RCT described above. The women’s sample size for the RCT was 462 pregnant women.

### Recruitment

Men were recruited into the study when they accompanied their spouses following a word of mouth or invitation card invite. The men were eligible as long as their partner had enrolled into the trial. We obtained informed consent from all men that accompanied their partners for PMTCT services. A demographic baseline questionnaire was administered on enrollment and also a questionnaire on the men’s involvement in PMTCT. The demographic data described in this article were obtained from the men that accompanied their partners for PMTCT services using a demographic questionnaire. The data describing the reasons for lack of participation of male partners in the PMTCT services were collected from the women, who reported to the study clinics on subsequent visits unaccompanied by their male partners.

### Ethical approval

The University of Malawi College of Medicine Research and Ethics Committee (COMREC) approved the protocol, consent forms and invitation card on 3 June 2013 (identifier: COMREC No P 09/12/1279). The Blantyre District Health Office permitted the conduct of the trial in the two health centres. All men provided a written informed consent (or a witnessed thumbprint if illiterate) prior to study participation. The trial was registered with Pan African Clinical Trials Registry www.pactr.org (Identifier: PACTR No 201311000675100).

### Data management

All data were captured in a Microsoft Access Database. Data were cleaned for completeness and incomplete information was deemed as missing with a designated code.

### Statistical analyses

Descriptive statistics were tabulated in Stata to compare the men that reported in the invitation card and word of mouth invites groups at baseline. We also present a summary of proportions highlighting the reasons for non-reporting by the men for the PMTCT services following the invitations. We present the interquartile range (IQR) while we summarized categorical variables using proportions.

## Results

Between June 2013 and February 2014, 993 pregnant women were screened for eligibility and 462 women were enrolled in the trial at a 1:1 ratio. Of the 462 women randomized, 109 (23.59%) women came back to the clinic with their male partner following the intervention. Of the 109 men who came, 65 (59.63%) were in the invitation card group while 44 (40.36%) were in the word of mouth group.

### Male participant’s baseline characteristics

Baseline characteristics of the men that reported to the clinic were comparable between the groups (Table 1). The median age of the male partners in the invitation card group was 29 years, interquartile range (IOR) 23-33 years while in the word of mouth invite group; it was 28.5 years, IQR 25-35 years. The majority of the male partners were educated to secondary school level with 30 (46.2%) in the invitation card group and 21 (47.7%) in the word of mouth invite group. About half of male participants in each arm were self-employed with 27 (41.5%) and 16 (36.4%) in the intervention and control arm respectively. Most men who reported to the clinic 77 (70.64%) reported to be HIV uninfected while 14 (12.84%) were HIV infected and 18 (16.51%) had an unknown HIV status (Table 2). Of the 14 HIV infected men, 9 were on ART while 5 had not initiated on ARVs because 2 were waiting for CD4 count results to determine eligibility for ARVs, one was scheduled for a counselling session and the other 2 never showed up for counselling after referral.

**Table 1:** Characteristics of male participants (*n*=109)

Characteristics	Intervention N=65	Control N=44	Total N=109	P Values
**Age (years)**				
Median (IQR)	29 (23-33)	28.5(25-35)	29 (23-34)	0.50
**Education**				
No education	4 (6.2)	2 (4.6)	6 (5.5)	
Primary	26 (40.0)	19(43.2)	45 (41.3)	
Secondary	30 (46.2)	21(47.7)	51 (46.8)	0.9
Tertiary	5 (7.7)	2 (4.6)	7 (6.4)	
**Employment Status**				
Not employed	15 (23.1)	13(29.6)	28 (25.7)	
Formally employed	23 (35.4)	15(34.1)	38 (34.9)	
Self employed	27 (41.5)	16(36.4)	43 (39.5)	0.67
**HIV Status**				
Negative	45 (69.2)	32(72.7)	77 (70.6)	0.61
Positive	10 (15.4)	4(9.1)	14 (12.8)	
Unknown	10 (15.4)	8(18.2)	18 (16.5)	

**Table 2:** Male partner’s reaction after receipt of an invitation

Partners Reaction	Intervention Arm (N=145)	Control Arm (N=162) n (%)	Total (N=307) n (%)	P Value
	N (%)	N (%)	N (%)	
Accepted	133 (91.72)	142 (87.65)	275/307 (89.58)	0.24
Declined Invitation	19 (13.10)	18 (11.11)	37 (12.05)	0.59
Angry with the Invite	3 (2.07)	1 (0.62)	4 (1.30)	0.26
Confused with the invite	0 (0)	1 (0.62)	1 (0.33)	0.34
Promised to attend	57 (39.31)	79 (48.77)	136 (44.30)	0.10
Did not say anything	6 (4.14)	8(4.94)	14 (4.56)	0.74

Note: Only relevant proportions have been presented, therefore the figures are not adding up to N

### Male partners’ reaction following invitation

Of the 462 women randomized, the majority, 353/462(76.41%) women returned to the clinic without their partners 165 (71.74%) and 188 (81.03%) from the invitation card group and the word of mouth invite group (standard of care group) respectively. Of the women that showed up without their male partners 307/353 (86.97%) had complete data that have been used in this section. Upon presenting the invitation, in total, 89.58% of women reported that their male partners accepted the accepted the invitation (Table 2). Majority of the women reported that their partners were neither angry nor confused with them being invited to the ANC clinic (Table 2). None of the women who reported that their partner was angry at the invitation reported of experiencing any physical abuse from her partner. In total, just below half (48.56%) of the women stated that their partners promised to accompany them at the next visit while 5% of the women stated that their partners did not comment or say anything following the invitation (Table 2).

### Reasons for not reporting for PMTCT services following an invitation

The major reason as reported by the women in their partners’ non-reporting was the non-availability of their male partners secondary to work obligations (Table 3). Seventeen percent of the women in each group reported that their male partners were not interested in accompanying their partners for the service while others promised their female partners that they will report at the next clinic visit or expressed other reasons for not reporting.

**Table 3:** Male partners’ reasons for not reporting at the clinic following an invitation

Reason	Intervention (n=145)	Control (n=162)	Total (n=307)	P Value
	N (%)	N (%)	N (%)	
Uninterested	26 (17.93)	27(16.67)	53(17.26)	0.77
Unavailability	116 (80.00)	135(83.33)	251(81.76)	0.45
Next Visit	118 (81.38)	134(82.72)	252(82.08)	0.6
**Other**	24 (16.55)	26 (16.05)	50 (16.29)	0.91

Note: Only relevant proportions have been presented, therefore the figures are not adding up to N

## Discussion

The characteristics of men that accompanied their partners for PMTCT were similar between the two groups, suggesting that the demographic aspects of the men may not be significant contributors to their attendance to the PMTCT services in this setting. The majority of the men were HIV uninfected while those that were HIV infected were linked to care and were either on ARVs or in Pre ART. This result underscores the opportunity that MI in PMTCT creates for HIV testing and linkage to care for men as reported in Burkina Faso [16] and a health education forum for men where health information is shared [4, 17, 18]. This result remains consistent with findings in Papua New Guinea where men who reported for PMTCT were linked to care after attending to PMTCT services [19] thereby highlighting other avenues for men to access HIV testing and antiretroviral therapy [20]. Although the invitation card was superior to the word of mouth-invite, the card was not a panacea solution and could not serve all male partners. There remain barriers to MI in PMTCT because most men accepted the invitation irrespective of the study group (invitation card versus word of mouth); however it did not translate in most men accompanying their partners. This finding remains congruent with what Tadesse et al [13] found in Blantyre, Malawi, that despite women believing that their partners would accompany them for antenatal care only 5.2% of the women were ever accompanied by their partners. This result emphasizes the urgency in finding complementary strategies that may be rolled out at the same time to increase MI in PMTCT services and also eliminating the barriers for MI. Despite our study being in a semi urban area, it concurred with what Jeffreys et al. in a Tanzanian implementation study concluded, that official invitation cards may be more effective for MI in PMTCT in a rural area and that the semi-rural areas may need more strategies beyond the letters to increase uptake of the service by men [21]. The non-availability of men secondary to work or other obligations was the main reason for lack of male involvement in this study. Similarly, a review on the barriers to MI in PMTCT services in sub-Saharan African by Morfaw et al. [22] highlighted time constraints secondary to socioeconomic demands and other obligations as a barrier to MI. The formative study prior to this trial alluded to this dilemma that results from work commitments, responsibility of fending for their families versus accompanying their partners for antenatal care [23]. This suggests that male partners may not accompany their partners not out of disinterest, but because of other competing interests such as providing for their families. This finding necessitates the strategies or policies that encourage employers to allow men to accompany their partners for PMTCT without negative consequences to the employees [24]. Notably, there were some men in this study who were not interested with involvement in the PMTCT services. The lack of interest expressed in this study concurs with findings by Auvinen et al. in a study conducted in Zambia where men were also disinterested with participation in the PMTCT programme [25, 26] and could be partially explained by the lack of clear policies on the role of men in the programme and the lack of inclusion of men from the inception of the programme [2, 27–31]. Furthermore, the cultural inappropriateness of MI in female dominated and centred programme [1, 32–34] explains the lack of action.

### Study strengths and limitations

The strength of this study is that it presents findings directly from men who reported for PMTCT services. These men may be different from the men that never reported as such the findings may not be generalizable beyond this group. The reasons for non-reporting were solicited from the women and not directly from the men themselves, which may potentially be biased but since they are partners to the men, they were the best proxy to interview.

## Conclusion

The characteristics of men that report for PMTCT services and the barriers to their involvement are critical in the development of male centred projects within the PMTCT programme. The men that reported were similar in the two groups, suggesting that demographic characteristics may not greatly influence their decision. There is need to develop more flexible strategies to include men in PMTCT programmes. There is need to consult with labour laws and act to ensure that men are protected from negative consequences when they accompany their partners for PMTCT services.

### What is known about this topic

- Demographic characteristics are associated with male attendance to PMTCT;
- Men have conflicting roles that hinder their attendance to PMTCT services;
- Men abuse their partners for attending to PMTCT.

### What this study adds

- Although men accepted an invitation to PMTCT, the majority did not report suggesting that an invitation alone is not enough for them to attend PMTCT services;
- Flexible and multiple strategies rolled out at the same time may improve male attendance to PMTCT services;
- There are some men who will not refuse nor accept an invitation to attend PMTCT service.
